# Effect of Waste PET Fiber on the Mechanical Properties and Chloride Ion Penetration of Emergency Repair Concrete for Road Pavement

**DOI:** 10.3390/ma17215352

**Published:** 2024-10-31

**Authors:** Su-Jin Lee, Hyungjin Shin, Han-Na Lee, Sang-Hyun Park, Hyoung-Moo Kim, Chan-Gi Park

**Affiliations:** 1 Department of Architectural Engineering, Keimyung University, Daegu 42601, Republic of Korea; sjlee@kmu.ac.kr; 2 Rural Research Institute, Korea Rural Community Corporation, Ansan 15634, Republic of Korea; shjin@ekr.or.kr; 3 Department of Regional Construction Engineering, Kongju National University, Yesan 32439, Republic of Korea; 20230243@smail.kongju.ac.kr (H.-N.L.); wnahd4989@naver.com (S.-H.P.); hyoungmoo@ex.co.kr (H.-M.K.)

**Keywords:** mechanical properties, durability, chloride ion penetration, waste PET fiber, pavement repair, latex-modified concrete

## Abstract

This study evaluated the effects of adding waste PET fibers on the mechanical properties and chloride ion penetration of latex-modified ultra-rapid hardening cement concrete used for emergency road pavement repairs. The primary experimental variable was the content of waste PET fibers. The mechanical properties of the concrete were evaluated through compressive strength, flexural strength, and splitting tensile strength tests. Its durability was evaluated through chloride ion penetration, surface resistivity, and abrasion resistance tests. The experimental results were compared with the quality standards for emergency repair concrete set by the Korea Expressway Corporation. As a result, this study has enhanced the strength and resistance to chloride ions of latex-modified concrete by incorporating waste PET fibers. In the mixture with 3.84 kg/m^3^ of waste PET fibers, the compressive strength was 29.9 MPa at 4 h and 42.5 MPa at 28 curing days. The flexural strength was 6.0 MPa at 4 curing hours and 7.0 MPa at 28 days, and the splitting tensile strength was 4.5 MPa at 28 days of curing. The chloride ion permeability amount and abrasion depth were 1081C and 0.82 mm, respectively. The mixture with 3.84 kg/m^3^ of waste PET fibers has superior compressive strength, flexural strength, splitting tensile strength, chloride ion penetration, and surface resistivity compared to the mixture with 7.68 kg/m^3^. This result means that the waste PET fibers caused poor dispersion and fiber-balling within the concrete, leading to loose internal void structures when incorporated at 3.84 kg/m^3^. However, the abrasion resistance test showed better results for the mixture with 7.68 kg/m^3^ of waste PET fibers than the 3.84 kg/m^3^ mixture. Therefore, the test results indicated that 3.84 kg/m^3^ of waste PET fibers is the most effective for latex-modified concrete used in emergency road pavement repairs.

## 1. Introduction

Concrete pavement is a method known for its high resistance to heavy vehicles and low maintenance costs and is one of the primary types of pavement used to ensure the long service life of highways in Korea [1]. As the service life of these pavements increases, the repair and reinforcement of aged concrete have become urgent. Considering the inconvenience to citizens and the challenges of traffic control, the repair of road pavement is typically conducted during early morning hours when traffic is minimal. In Korea, ultra-rapid hardening cement is used for this purpose. However, this type of cement, while achieving early strength development, tends to produce higher hydration heat than Type I cement, making it prone to microcracking. These microcracks can occur within the concrete, increasing permeability and subsequently reducing durability.

Repair materials applied to concrete structures such as roads and railways mainly include polymer-based acrylics and latexes. However, there are concerns about quality deterioration due to the increased cost of repairs [2]. Recently, an ultra-thin-continuously reinforced concrete pavement (UT-CRCP) has been developed for repairing aged cement concrete pavements [3]. This method is designed to overcome the limitations of existing repair methods by installing continuously reinforced concrete pavement (CRCP) over jointed plain concrete pavement (JPCP). Additionally, Tianxiong Guo et al. [4] evaluated the fatigue characteristics of precast concrete repair methods for road pavement repairs. The evaluation results showed that these methods provided excellent support to the upper structure in both static incremental loading tests and fatigue loading tests. Furthermore, one way to address the issue of cracks in concrete caused by high hydration heat is the application of reinforcing fibers.

These fibers are known to significantly control the growth of microcracks in concrete members by increasing the tensile strength between the binders [5]. A wide variety of synthetic polymer fibers are deemed suitable for application in FRC, including but not limited to polypropylene (PP), nylon, polyvinyl alcohol (PVA), polyolefin (PO), carbon, polyethylene (PE), polyester (PET), acrylic (PAN), and aramid [6]. Among these, steel fibers are the most commonly used reinforcement material. However, it presents challenges due to its high rigidity and weight, which can cause wear and tear on pumping hoses and create safety management difficulties. Additionally, issues such as durability degradation from steel fiber corrosion and high rebound loss need to be addressed [7].

Structural synthetic fibers have been developed and applied as an alternative to overcome the drawbacks of steel fiber-reinforced concretes [8]. Additionally, recent research has been actively exploring the use of reinforcing fibers made from waste PET (Polyethylene terephthalate) bottles to improve the properties of concrete. As the use of PET bottles has increased, research has been conducted to recycle them, and their material properties have been considered for application as concrete reinforcing fibers. Utilizing waste PET bottles as concrete reinforcing fibers can be a highly beneficial method from an environmental perspective [9,10,11]. Studies on the application of waste PET fibers in concrete include the following: Alani et al. [12] concluded that adding PET fibers with a high aspect ratio at 1% to ultra-high-performance green concrete containing ultrafine palm oil fuel ash and silica fume increased flexural strength by 18%. In more detail, its mechanical properties include a compressive strength of 153 MPa, splitting tensile strength of 9.31 MPa, flexural strength of 30.0 MPa, flexural toughness of 29.7 kN·mm, and flexural stiffness of 18.225 kN/mm, achieving a significant increase in the ductility index. This confirmed that adding waste PET fibers can enhance the stiffness and reduce the brittle behavior of ultra-high-performance concrete. Additionally, it was evaluated whether the PET fiber reinforcement was incorporated into the concrete and changed its short-term and long-term performance. It also examined the performance degradation of waste PET fiber reinforcement when exposed to acidic and alkaline environments. The results of the exposure tests showed that the strength retention rate ranged from 83.4% to 96.4% in acidic environments, while in alkaline environments, it ranged from 42.4% to 97.9%. This means that the strength retention rate of the fiber itself decreases significantly when exposed to strong alkaline conditions at a high temperature, whereas it increases in processed fibers. In the case of PET fiber-reinforced concrete mixes, the flexural strength and equivalent flexural strength developed stably over time; therefore, it was concluded that there is no performance degradation due to hydrolysis concerns associated with the use of PET fibers [13].

PET is a plastic material widely used in various products, such as beverage containers [10]. Additionally, the PET synthetic fiber reinforcement exhibits high tensile strength, unlike the commonly used PE fibers and PP fibers overseas, and does not corrode like steel fibers, making it easier to store [11]. Studies on various reinforcing fibers have been conducted to improve the performance of concrete pavement repair [14,15,16,17]. Among them, steel fibers can cause corrosion concerns when a deicing agent is used to remove snow from the road pavement in winter. Therefore, it is necessary to use synthetic fibers that do not have the risk of corrosion [18,19,20,21,22,23]. In the case of synthetic fibers, polypropylene fibers are usually used, but there is a disadvantage in that dispersibility in concrete becomes less due to their low density [10,13,14]. Therefore, it is necessary to apply a fiber reinforcement material capable of solving this problem to road pavement repair, and accordingly waste PET fibers are used in this study. In addition, using waste PET fibers as a road pavement repair material has advantages in the environmental aspects presented above [5,8,10,11,12,13].

This study evaluated the effectiveness of waste PET fibers as a reinforcement in improving the crack control, strength, and chloride ion penetration resistance of latex-modified concrete for emergency road repairs. For this purpose, performance changes were tested and analyzed through the amount of waste PET fibers.

## 2. Experimental

### 2.1. Materials and Mix Proportions

The cement used was ultra-rapid hardening cement (GmaxRapid Co., Ltd., Namyangju, Republic of Korea); its chemical composition is shown in Table 1. The coarse aggregate consisted of crushed stones with a maximum size of 13 mm, and the fine aggregate consisted of river sand with a specific gravity of 2.60, fineness modulus of 6.92, and water absorption of 0.35%. For latex, styrene-butadiene latex (SB latex, Jungang Polytech Co., Ltd., Pyeongtaek, Republic of Korea) was used; its main components are shown in Table 2. SB latex enhances fluidity by facilitating interactions among surfactants and other components, particularly at low W/Cs. Additionally, when a latex film forms, it improves the bonding strength between materials, which decreases water permeability and enhances mechanical properties. The workability of the mixture is further improved without increasing the W/C, thereby reducing the risk of cracking caused by concrete shrinkage [14,15]. However, it is important to note that the inclusion of latex in concrete may delay the initial strength development [16].

**Figure 1:**
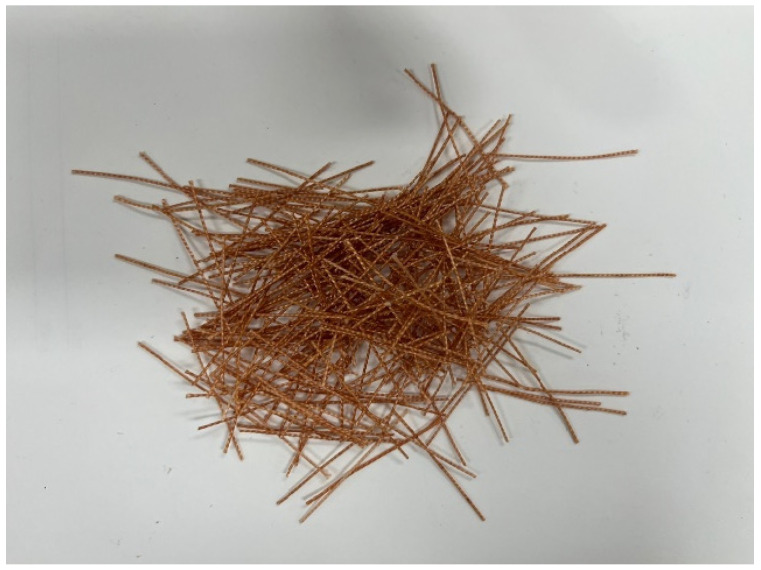
Shape of waste PET fiber.

PET fiber is used as a reinforcement (Contech ENG Co., Ltd., Yongin, Republic of Korea); its properties are shown in Table 3, and the shape of the fibers is shown in Figure 1.

The mechanical properties and chloride ion penetration of concrete using waste PET fibers were assessed for pavement repair material according to the standards of the Korea Expressway Corporation [24]. For repairing concrete pavement using ultra-rapid hardening cement, organizations such as AASHTO, various U.S. state Departments of Transportation [25], and the Korea Expressway Corporation specify a minimum curing time of 4 h before opening to traffic. The traffic opening criteria also include mechanical properties: 21 MPa for compressive and 3.5 MPa for flexural strength. Additionally, the 28-day strength requirements are specified: 35 MPa for compressive strength, 4.5 MPa for flexural strength, and 4.2 MPa for splitting tensile strength. Therefore, in this study, ultra-rapid hardening cement was employed as the concrete pavement repair material, and the target properties were decided in accordance with the above-mentioned regulations.

The mix proportions consist of a water–cement ratio of 28%, a unit cement weight of 400 kg/m^3^, and latex content at 10.4% of the unit weight of cement based on solid content.

The latex used in this study consists of a liquid material containing 49% solid content and 51% water. In general, the amount of latex added to latex-modified concrete is determined by the ratio of solid content to the binder. For this study, approximately 10% of the solid content was utilized for the latex [14,15]. A total of 112 kg/m^3^ was added, which includes the amount of water incorporated into the liquid latex, resulting in a W/C of about 28%. The waste PET fibers used in the study were applied at three different quantities: 0.00 kg/m^3^, 3.84 kg/m^3^, and 7.68 kg/m^3^. The mix proportions examined in this study are detailed in Table 4. The mixing ratio incorporates waste PET fiber as a variable in the combination used for highway pavement by Contech Eng. Co., Ltd. and Sanbong Eng. Co., Ltd. in the Republic of Korea.

### 2.2. Test Methods

#### 2.2.1. Compressive Strength Tests

**Figure 2:**
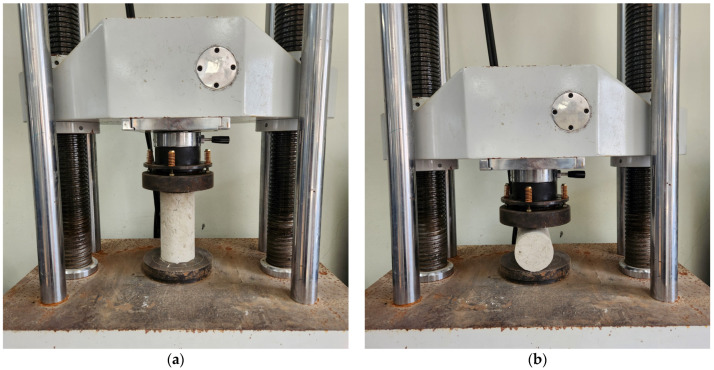
Setup of strength test under the compressive and splitting tensile strength test. (**a**) Compressive strength test; (**b**) Splitting tensile strength test.

The compressive strength test was conducted using a cylindrical specimen sized Ø100 × 200 mm. A total of six specimens were made for each curing age and were demolded after 4 h. The tests were performed at curing ages of 4 h, 7 days, and 28 days. The testing environment maintained a temperature of 20 ± 2 °C and a relative humidity of 65% or higher, according to ASTM C39/C39M [26]. The compressive strength was measured at a load rate of 0.25 ± 0.05 MPa/s and is reported as the average compressive strength. Figure 2a shows the setup for the compressive strength test.

#### 2.2.2. Splitting Tensile Test

Splitting tensile tests were conducted in accordance with the ASTM C496 [27] by using cylindrical specimens of the same size as compressive strength test one. A total of six specimens were made for each curing age and were demolded after 4 h. The tests were performed at curing ages of 4 h, 7 days, and 28 days (Figure 2b). The splitting tensile strength was measured at the same load rate under the same environmental conditions as the compressive strength test.

#### 2.2.3. Flexural Tests

**Figure 3:**
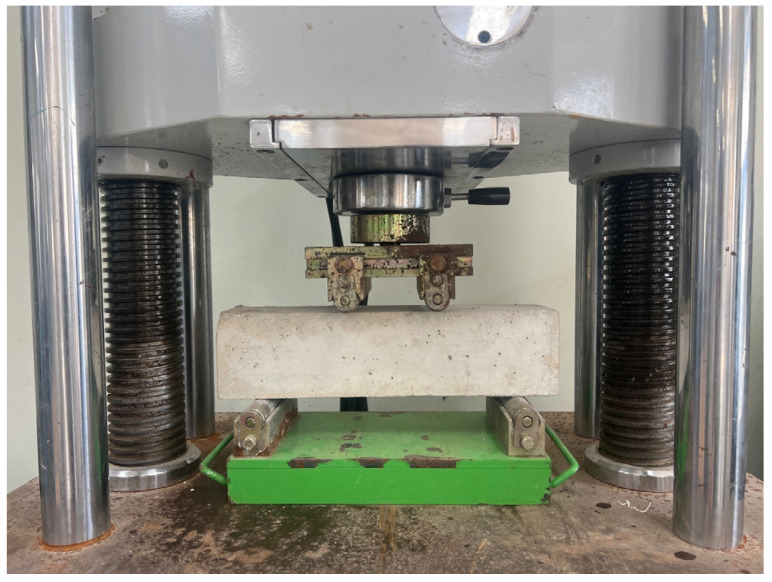
Flexural strength test setup.

The flexural strength test was conducted using prismatic specimens sized 100 × 100 × 400 mm. The specimens were demolded after 4 h and tested at curing ages of 4 h, 7 days, and 28 days. The tests were performed by ASTM C78/C78M-22 [28]. The experimental environment maintained a temperature of 20 ± 2 °C and a relative humidity of 65% or higher. Figure 3 shows the flexural strength test setup. The flexural strength test was carried out at a load rate of 0.25 ± 0.05 MPa/s and was presented as the average strength value for six test specimens.

#### 2.2.4. Chloride Ion Penetration Tests

The evaluation of chloride ion penetration, which significantly impacts the service life of concrete pavements, was conducted using the ASTM C1202-19 test method [29]. According to the standards set by the Korea Expressway Corporation [24], the target value for charge passed was established at 2000 Coulombs or less after 7 days. The experimental conditions were controlled to maintain a temperature of 20 ± 2 °C and a relative humidity of 65% or higher. Cylindrical specimens sized Ø100 × 200 mm were demolded after 4 h. At 7 days, the specimens were cut to a thickness of 50 mm at their center, following the Korea Expressway Corporation’s standards, before the chloride ion penetration test was conducted.

**Figure 4:**
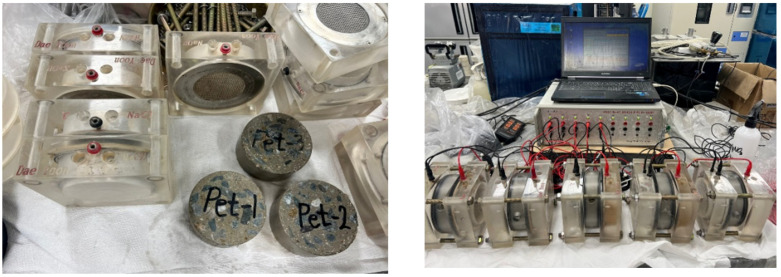
Setup of Chloride ion penetration test.

The cut specimens were in a desiccator and completely sealed, and a vibration pump was operated for 3 h to remove the entrapped air inside the specimen. Water was then poured to a level that submerged the specimen, and the pump was operated for an additional hour. After stopping the vacuum pump, the specimens were fully saturated by immersing them in water for 18 ± 2 h. The specimens were then fixed using an A.V. Cell, commonly used for chloride ion penetration resistance tests. A 3.0% NaCl solution was added to the cathode, and a 0.3 N NaOH solution was added to the anode. The A.V. Cell was then connected to a DC power supply set at 60 ± 0.1 V, and the current values were recorded for 6 h. This study’s target value was 2000 Coulombs or less for charge passed. The chloride ion penetration based on the charge passed, as suggested in ASTM C1202-19, is shown in Table 5, and Figure 4 shows the chloride ion penetration test setup. The test results were presented as the average value for six test specimens.

#### 2.2.5. Surface Resistivity

**Figure 5:**
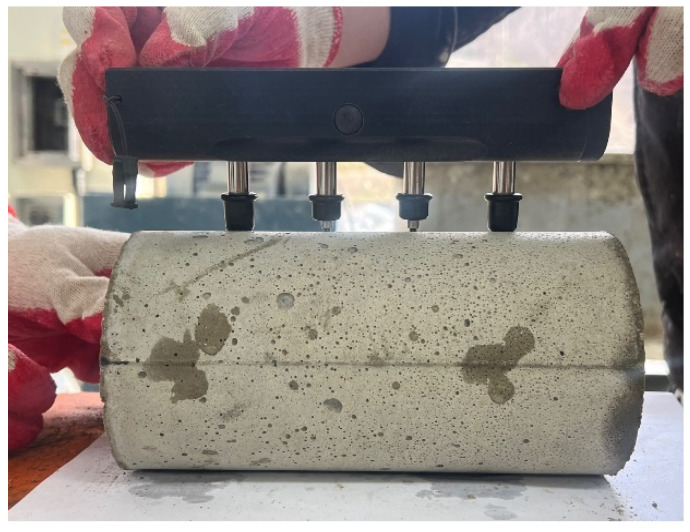
Surface electrical resistivity test setup.

Concrete’s durability is lost, and rebar corrosion occurs due to chloride ions, which reduce the (electrical) resistivity of the concrete. Therefore, the surface (electrical) resistivity test of the concrete was conducted according to AASHTO T358 [25]. The experimental conditions were controlled to maintain a temperature of 20 ± 2 °C and a relative humidity of 65% or higher. Specimens for each mix were prepared and demolded after 4 h, followed by a 7-day curing period. The chloride ion penetration ratings are listed based on electrical resistivity values in Table 5, and Figure 5 shows the concrete (electrical) resistivity test setup.

Following the AASHTO TP 95-14 test method [25], cylindrical specimens sized Ø100 × 200 mm were prepared for each mix. The surface resistivity of the specimens in a dry condition was measured using the Four-Point Wenner Probe method. The test results were compared and analyzed with the chloride ion penetration test results. Additionally, the test results were presented as the average value for six test specimens.

#### 2.2.6. Abrasion Test

**Figure 6:**
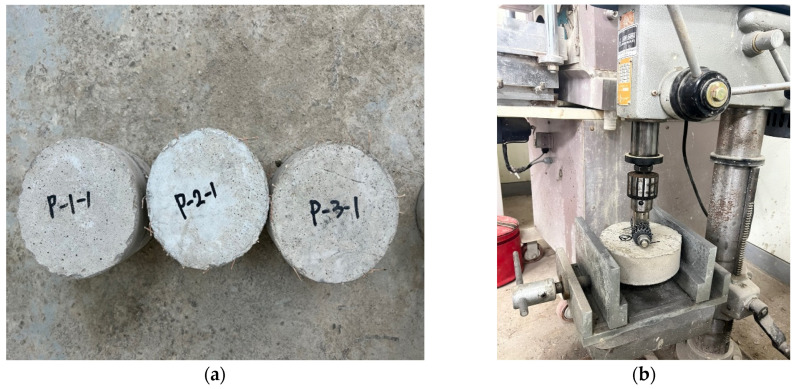
Abrasion test setup. (**a**) Abrasion specimens; (**b**) Test set-up.

According to the Korea Expressway Corporation standards, the abrasion test should meet 2 mm for the depth of wear. The abrasion test was conducted following ASTM C944-99 [30], specifically Procedure B. The experimental conditions were controlled to maintain a temperature of 20 ± 2 °C and a relative humidity of 65% or higher. For the abrasion test, specimens were prepared for each mix, demolded after 4 h, and cured for 7 days. The test was conducted in two stages: initially for 15 min, after which the powder from the specimen was removed, and then for an additional 15 min. The test results were presented as the average value for six test specimens. Figure 6 shows the abrasion test setup.

## 3. Test Results

### 3.1. Compressive Strength

**Figure 7:**
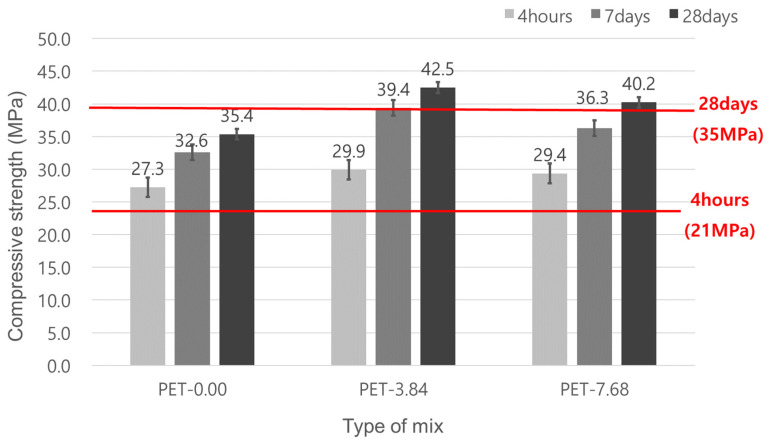
Compressive strength of emergency repair concrete for pavement.

The compressive strength test results are shown in Figure 7. The results indicate that the target strengths of 21 MPa at 4 h and 35 MPa at 28 days were met for all mixes. According to previous research, the initial strength for emergency repair concrete met the target compressive strength of 21 MPa regardless of the curing age for all mixes. Generally, for latex-modified repair concrete, latex has a strength-retarding effect, requiring an increase in the amount of rapid-hardening cement to achieve initial strength. In this study, rapid-hardening cement employed 400 kg/m^3^ to ensure initial strength. This amount of rapid-hardening cement is commonly applied in conventional road repair concrete. However, compared to previous results, this study showed an increase in initial strength. This is because the previous research typically used about 15% latex by weight of cement [17], while this study reduced the latex content to 10.4%, thus reducing the strength-retarding effect.

For the impact of the waste PET fiber content, test results showed an increase in compressive strength with curing age compared to the PET-0.0 mix, Additionally, the PET-3.84 mix has a higher strength than the PET-7.68 mix This result is caused by the high quantity of waste PET fibers, which leads to insufficient dispersion in the concrete. This insufficient dispersion causes fiber balling and increases voids, ultimately decreasing strength [10,14,15,16,17,19,20,21].

Several factors affect the dispersibility of fibers [10,19,20,21,31,32,33,34,35]. These factors include the fiber’s surface characteristics, aspect ratio, volume fraction, length, density, and geometry [10,19,20,21,31,32,33,34,35]. Additionally, numerous studies have been conducted to enhance the dispersibility of fibers in concrete. For instance, CNTs have well-known poor dispersibility in the cement matrix. A study to improve this dispersibility was conducted by applying (1) dry mixing, (2) ball milling, and (3) ultrasonic methods as a method to properly disperse CNTs in cement matrix [34]. In addition, a study to improve the dispersibility of CNTs was conducted to evaluate the effect of the length, diameter, and concentration of CNTs. The study determined the optimal dimension and concentration of CNTs that could improve the performance of cement composite materials [35].

As in the above study, determining the dispersibility of fibers is very important in improving the performance of concrete. In this study, all other conditions were the same, and only the amount of fiber added was changed. Therefore, the amount of fiber added also affects the performance of concrete. In particular, when the amount of fiber added increases, the workability decreases because phenomena such as the bridging effect and pullout of the fiber occur. Decreasing workability can reduce the dispersibility of fibers and increase the voids in concrete, thereby reducing performance [14,15,16,17,19,20]. In this study, the mixing time was set to 90 s after adding fiber reinforcements, and this was applied equally to the mixes without fiber reinforcement (90 s mixing time after all materials were added). Additionally, the amount of latex was reduced from 15% to 10.4% compared to conventional mix proportions. As a result, since the same mixing method was applied to improve dispersibility, PET-7.68, which has a relatively high mixing amount, tended to decrease compressive strength compared to PET-3.84.

### 3.2. Flexural Strength

**Figure 8:**
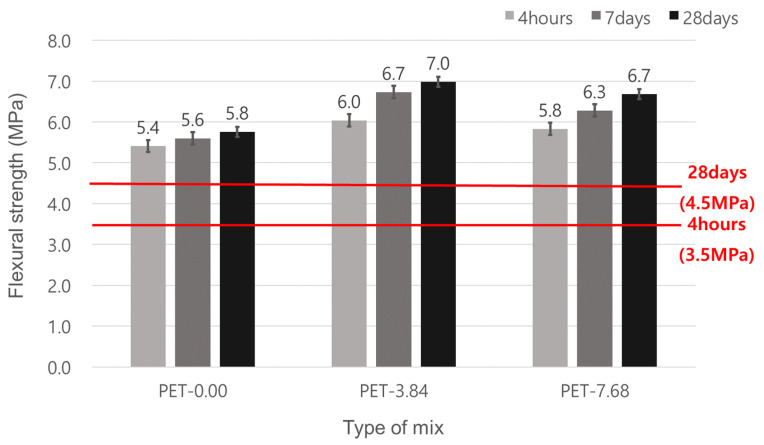
Flexural strength of emergency repair concrete for pavement.

The flexural strength test results for the mixes with waste PET fibers showed that all mixes met the target flexural strength of 3.5 MPa at 4 h and 4.5 MPa at 28 days (Figure 8). The waste PET fiber-reinforced concrete has superior flexural strength compared to the non-reinforced emergency repair concrete. Waste PET fibers can enhance the strength of concrete as structural fibers.

For fiber content, the PET-3.84 mix has a higher flexural strength than the PET-7.68 mix. This result indicates that the dispersion of fibers was more effective in the 3.84 kg/m^3^ content, whereas, at the 7.68 kg/m^3^ content, fiber balling increased voids, slightly reducing the flexural strength. Previous studies have shown that a higher fiber content can lead to fiber clumping, reducing overall strength [15,16,17,19,20]. Therefore, at a 7.68 kg/m^3^ waste PET fiber content, improving fluidity and increasing mixing time are necessary to enhance fiber dispersion.

### 3.3. Splitting Tensile Strength

**Figure 9:**
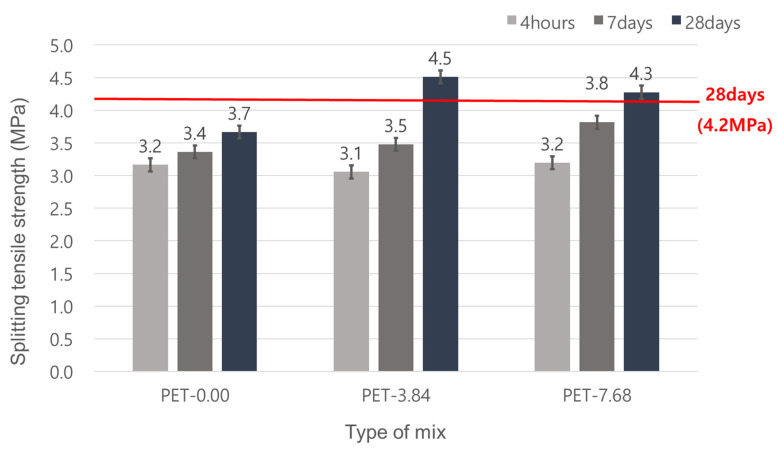
Splitting strength of emergency repair concrete for pavement.

In this study, the target splitting tensile strength was set to 4.2 MPa at 28 days, following the standards of the Korea Expressway Corporation. The experimental results indicated that adding waste PET fibers increased the splitting tensile strength (Figure 9). The mix without waste PET fibers (PET-0.0) showed an increase in splitting tensile strength with age but failed to meet the target strength, achieving only 3.67 MPa at 28 days.

The test results showed that PET-3.84 has a higher splitting tensile strength than PET-7.68 Additionally, the splitting tensile strength of all mixes increased with age. At 28 days, the splitting tensile strengths of the mixes with 3.84 kg/m^3^ and 7.68 kg/m^3^ waste PET fiber content were 4.51 MPa and 4.28 MPa, respectively.

Generally, structural fibers enhance the mechanical properties of concrete, such as tensile strength. The waste PET fibers used in this study also acted as structural fibers, improving the mechanical properties of the concrete, including tensile strength. Therefore, reinforcing waste PET fibers effectively increased the splitting tensile strength.

However, the splitting tensile strength of the mix with 7.68 kg/m^3^ waste PET fiber was lower than that of the mix with 3.84 kg/m^3^ fiber. This reduction is attributed to the decreased dispersion of fibers, which is crucial for the performance of fiber-reinforced concrete. There is a need for methods for increasing mixing time and enhancing fluidity to improve fiber dispersion.

### 3.4. Chloride Ion Penetration and Surface Resistivity

**Figure 10:**
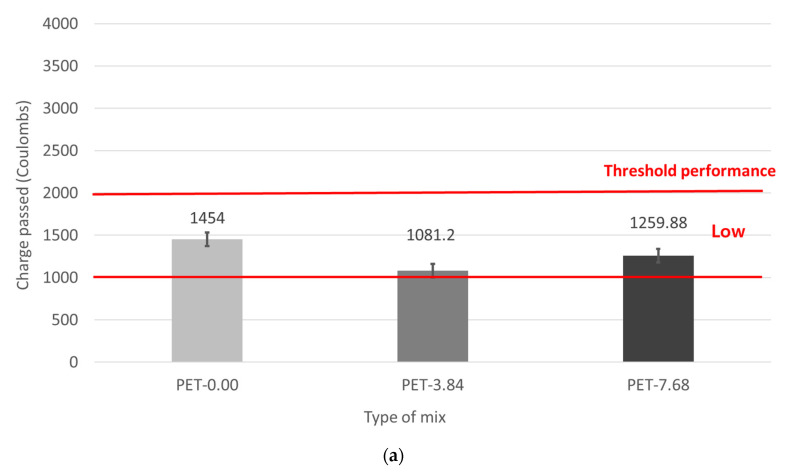

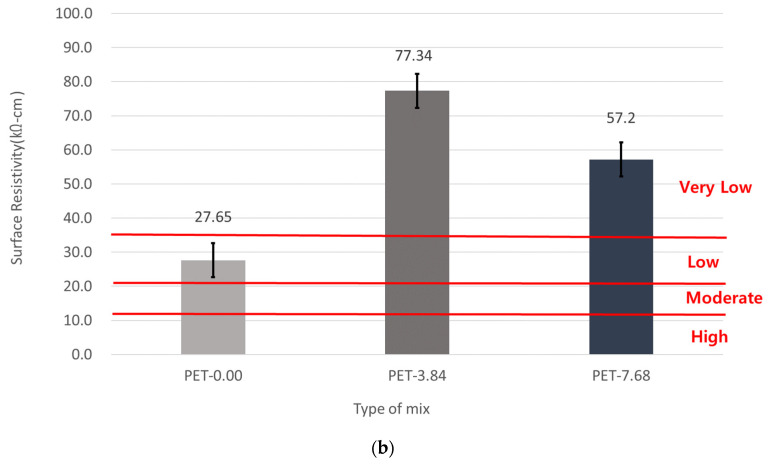
Permeability of emergency repair concrete. (**a**) Permeability rating based charge passed; (**b**) Permeability rating based surface resistivity.

The results of the chloride ion penetration resistance test according to the added amount of waste PET fibers are shown in Figure 10a. The Korea Expressway Corporation has regulated 2000 Coulombs or less for chloride ion penetration resistance for emergency repair concrete using rapid-hardening cement. As a result, whether with/without fiber, all mixes met the standard of 2000 Coulombs or less, which satisfied the ASTM permeability standard, indicating a “Very Low” permeability rating. The mix without waste PET fibers measured 1454 Coulombs, while the mixes with 3.84 kg/m^3^ and 7.68 kg/m^3^ additions of waste PET fibers measured 1081.5 Coulombs and 1259.9 Coulombs, respectively. This can be attributed to the latex used in this study, which is a polymer material that forms a latex film, thereby reducing the permeability of the concrete.

Previous studies also report that the rapid-hardening cement used in emergency repair concrete can increase permeability due to cracks caused by high initial hydration heat [14,15,16,17]. However, the addition of latex reduces permeability by forming a latex film [14,15,16,17]. When waste PET fibers were added, the chloride ion penetration decreased. This is because the fibers control the occurrence and growth of cracks within the concrete. Waste PET fiber decreases water permeability in concrete by regulating water movement through internal microcracks. It achieves this by suppressing the formation and growth of these cracks through various mechanical behaviors, such as the fiber bridging effect, debonding, pullout, and fiber fracture [5,8,10,11,12,13].

Furthermore, the chloride ion penetration was higher at 7.68 kg/m^3^ of waste PET fibers compared to 3.84 kg/m^3^. Poor dispersive concrete can cause fiber balling, which creates excess voids and increases permeability. Thus, the chloride ion penetration, which indirectly evaluates the permeability of concrete, was higher at 7.68 kg/m^3^ of waste PET fibers than at 3.84 kg/m^3^.

The surface electrical resistivity test is a way to indirectly evaluate the permeability of concrete based on chloride ion penetration classifications (Table 5). The experimental results are shown in Figure 10b. The surface resistivity test results indicated that the mix without waste PET fibers (PET-0.00) had an average value of 27.65 kΩ-cm, corresponding to a “Low” permeability based on chloride ion penetration standards. In contrast, the mixes with waste PET fiber showed resistivity values of 77.34 kΩ-cm for PET-3.84 and 57.2 kΩ-cm for PET-7.68, respectively, indicating “Very Low” permeability based on chloride ion penetration standards. Therefore, it was evident that adding waste PET fibers improves the permeability resistance of emergency repair concrete for road pavements. Additionally, similar to the chloride ion penetration results, the surface resistivity was significantly higher for the mix PET-3.84 compared to the mix PET-7.68.

### 3.5. Abrasion Resistance

**Figure 11:**
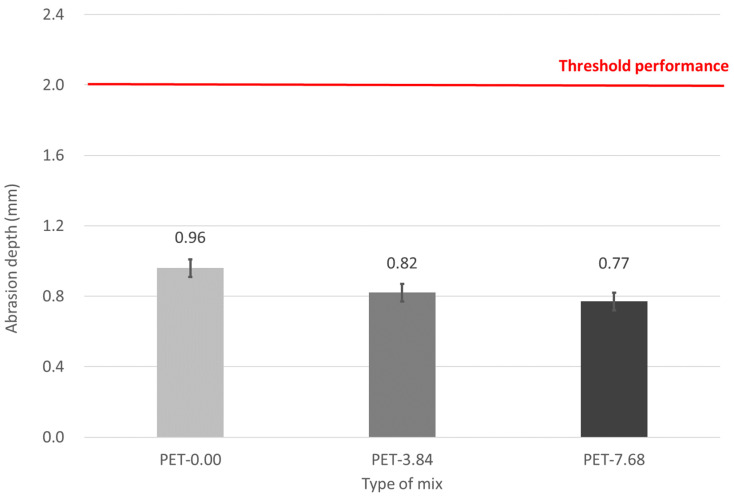
Abrasion depth of emergency repair concrete.

**Figure 12:**
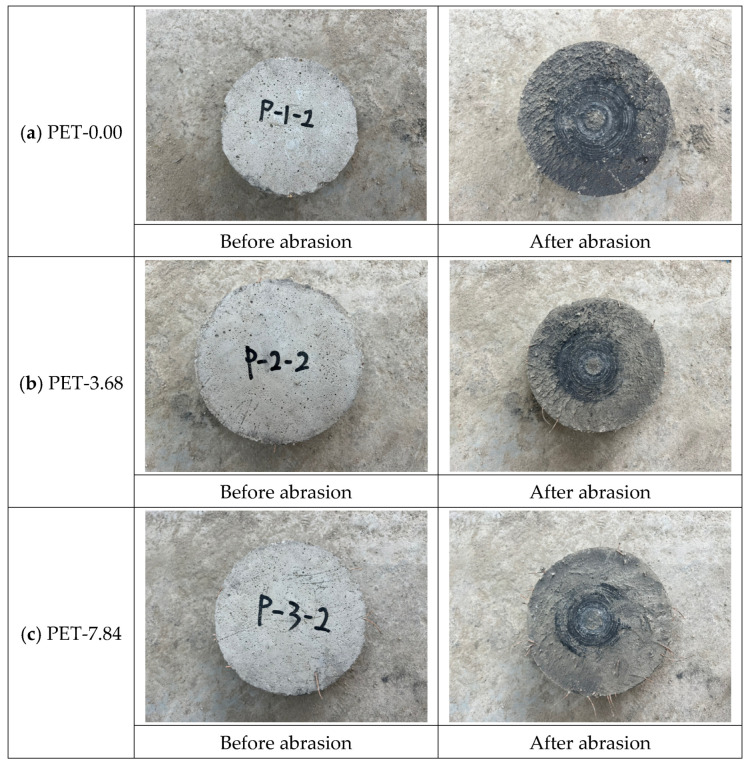
Comparison of the surface before and after the abrasion test.

The results of the abrasion (resistance) tests according to the added amount of waste PET fibers are shown in Figure 11. Regardless with/without fiber, all mixes met the Korea Expressway Corporation standard of 2 mm or less for the depth of wear. For adding waste PET fiber, the mixes with waste PET fibers reduce abrasion loss compared to the mix without fiber. This outcome can be attributed to the waste PET fibers densifying the concrete surface, thereby enhancing abrasion resistance. Furthermore, the abrasion resistance is enhanced with higher addition amounts of waste PET fibers. Waste PET fibers enhance the wear resistance of concrete by improving its mechanical properties through several mechanisms, including fiber bridging, de-bonding, pullout, and fiber fracture. These actions help to prevent the falling off of concrete particles, resulting in a more durable surface [10,13,14,15,16,17,20]. Figure 12 shows the concrete surface before and after the abrasion test. For the mix without fibers, the abrasion loss was relatively high. As the addition amount of waste PET fibers increased, the number of exposed fibers on the surface also increased. The greater exposure of fibers on the surface indicates that the fibers effectively prevent the dislodging of concrete particles during abrasion, thereby reducing abrasion loss. Thus, it can be concluded that adding waste PET fibers is effective in enhancing abrasion resistance.

## 4. Conclusions

In this study, the strength characteristics, permeability, and abrasion resistance of emergency repair concrete for road pavements with added waste PET fibers were evaluated. The experiments were conducted by varying the addition amounts of waste PET fibers. Overall, the incorporation of waste PET fibers in emergency repair concrete for road pavements proved to be beneficial in enhancing the material’s compressive strength, flexural strength, splitting tensile strength, permeability resistance, and abrasion resistance. These findings suggest that waste PET fibers can be effectively used to improve the performance and durability of concrete in road repair applications. The test results are summarized below.

1. All mixes met the compressive strength target values of 21 MPa at 4 h and 35 MPa at 28 days. The concrete mixes with waste PET fibers have superior compressive strength compared to the mix without fibers. The mix with 3.84 kg/m^3^ waste PET fibers showed a higher compressive strength compared to the mix with 7.68 kg/m^3^. This means that the mix with a lower amount of fiber has better dispersion effects than the mix with a higher amount.
2. For flexural strength, the concrete with waste PET fibers has a higher flexural strength than the concrete without fiber. The mix with 3.84 kg/m^3^ waste PET fibers showed a higher flexural strength compared to the mix with a 7.68 kg/m^3^ addition amount, again because of the improved dispersion effects of the fibers at the lower addition amount.
3. The mix without waste PET fibers did not meet the target strength of 4.2 MPa at 28 days. In contrast, the mixes with waste PET fibers did meet the target strength. The mix with a 3.84 kg/m^3^ addition amount of waste PET fibers showed better tensile strength than the mix with a 7.68 kg/m^3^ addition amount.
4. For permeability, two different tests were carried out: chloride ion penetration and surface electrical resistivity. However, these were classified as the permeability of concrete based on the charge passed equally. Regardless of whether with/without waste PET fiber, all mixes met the target values of 2000 Coulombs or less for charge passed, which is rated “Low” permeability. The mixes with waste PET fibers showed superior resistance to chloride ion penetration. Surface (electrical) resistivity tests indicated that the mixes with waste PET fibers had a “Very Low” permeability, confirming improved permeability resistance with the incorporation of waste PET fibers.
5. The mixes with waste PET fibers showed improved abrasion resistance. The mix with a 7.68 kg/m^3^ addition amount had the best abrasion resistance. This is because of the increased exposure of fibers on the concrete surface, which prevents the dislodging of concrete particles during abrasion.

Therefore, based on the comprehensive evaluation of mechanical properties and chloride ion penetration, it is concluded that a 3.84 kg/m^3^ inclusion amount of waste PET fibers is the most effective for latex-modified concrete used in emergency road pavement repairs. The 3.84 kg/m^3^ inclusion rate provides the best balance of compressive strength, flexural strength, splitting tensile strength, and resistance to chloride ion penetration, while also ensuring adequate abrasion resistance. This optimal inclusion amount ensures the enhanced performance and longevity of the repaired pavement, making it a practical recommendation for emergency road repair projects. To effectively apply the results of this study to road pavement in real-world settings, it is necessary to conduct an economic analysis regarding the use of waste PET fiber as well as an assessment of carbon emissions from an environmental perspective. Additionally, to evaluate long-term durability, further studies should be undertaken, including tests for repeated freezing–thawing cycles, resistance to concrete surface scaling, and adherence to chemical environments, such as exposure to chloride ions. This study also experimented with varying the amount of waste PET fiber added to the current mixing ratios used for road pavement repairs from a practical application standpoint. Consequently, additional research is needed on facilities or methods for incorporating waste PET fiber in field applications to ensure practicality.

## Figures and Tables

**Table 1:** Chemical composition of rapid set cement.

SiO_2_ (%)	Al_2_O_3_ (%)	Fe_2_O_3_ (%)	CaO (%)	MgO (%)	K_2_O (%)	SO_3_ (%)
13 ± 3	17.5 ± 3	3>	50 ± 3	2.5>	0.21	14 ± 3

**Table 2:** Properties of styrene butadiene latex.

Solids Content (%)	Styrene Content (%)	Butadiene Content (%)	pH	Specific Gravity	Surface Tension (dyne/cm)	Particle Size (A)	Viscosity (cps)
49	34 ± 1.5	66 ± 1.5	11.0	1.02	0.35	1700	642

**Table 3:** Properties of waste PET fiber.

Elastic Modulus (GPa)	Density (g.mm^3^)	Fiber Length (mm)	Fiber Diameter (mm)	Tensile Strength (MPa)	Aspect Ratio (L/D)
10	1.34	41.55	0.693	831.3	60

**Table 4:** Mix proportions of emergency repair concrete for pavement.

No. of Mix	G_max_ (mm)	W/C (%)	Unit Weight (kg/m^3^)								
			W			C	S	G	Latex		Waste PET Fiber
			Latex	Adding	Total				Solid	Water	
PET-0.00	13	28	43.35	68.65	112	400	767	839	41.65	43.35	-
PET-3.84											3.84
PET-7.68											7.68

**Table 5:** Criteria for Chloride Ion Penetrability based on charge passed and surface resistivity.

Chloride Ion Penetrability	Charge Passed (Coulombs)	Surface Resistivity (kΩ-cm)
High	>4000	<12
Moderate	2000~4000	12~21
Low	1000~2000	21~37
Very Low	100~1000	37~254
Negligible	<100	>254

## Data Availability

The original contributions presented in the study are included in the article, further inquiries can be directed to the corresponding author.
